# Preoperative inferior vena cava-abdominal aorta ultrasound examination to guide the positioning of spinal anesthesia to reduce post-spinal hypotension: a prospective, randomized trial

**DOI:** 10.3389/fmed.2025.1641899

**Published:** 2025-10-09

**Authors:** Haixing Wu, Ting Chen, Xiyuan Xie, Jing Ning, Yupeng Han, Suren Rao Sooranna, Qiuxia Huang, Han Wu, Rong Lin, Hailan Xue, Liang Lin, Xiaodan Wu

**Affiliations:** ^1^Department of Anesthesiology, Shengli Clinical Medical College of Fujian Medical University, Fuzhou, China; ^2^Department of Anesthesiology, Fuzhou University Affiliated Provincial Hospital, Fuzhou, China; ^3^Department of Ultrasonography, Fujian Provincial Hospital, Fuzhou, China; ^4^Department of Metabolism, Digestion and Reproduction, Imperial College London, Chelsea and Westminster Hospital, London, United Kingdom; ^5^Life Science and Clinical Research Center, Youjiang Medical University for Nationalities, Baise, China; ^6^Department of Gynaecology and Obstetrics, Fujian Provincial Hospital, Fuzhou, China; ^7^Department of Ultrasonography, Fujian Maternal and Child Healthcare Hospital, Fuzhou, China

**Keywords:** cesarean delivery, hypotension, IVC:Ao diameter, left uterine displacement, spinal anesthesia

## Abstract

**Background:**

The effectiveness of a 15° left-lateral tilt for alleviating the inferior vena cava (IVC) compression caused by a gravid uterus has been questioned. This study assessed the benefits of conducting pre-spinal anesthesia IVC-abdominal aorta ultrasound examinations and utilizing the IVC to abdominal aorta (IVC/Ao) ratio as a guide for post-spinal anesthesia positioning.

**Methods:**

200 parturients undergoing cesarean section were randomized 1:1 into an ultrasound-guided and a control group (groups U and C, respectively). The parturients in group C were positioned with a 15° left-lateral tilt, while those in group U had the operating table tilted to achieve the maximum IVC:Ao diameter, as determined by preoperative ultrasound, which indicated the maximum IVC:Ao diameter. The primary endpoint was the incidence of hypotension in parturients, defined as the period from the completion of intrathecal drug injection (time A) to when they were placed in the supine position (time B). Secondary outcomes included total vasopressor use, umbilical cord blood parameters (such as the pH and base excess values of the umbilical artery) immediately after birth, and Apgar scores at 1 and 5 min for the neonate.

**Results:**

The incidence of hypotension from the end of spinal anesthesia until the supine position was lower in group U (60.8%, *n* = 79) compared to group C (80%, *n* = 80) for the parturients included in the statistical analysis (risk difference = −0.192 (95% CI −0.325 to −0.050), *p* = 0.010). Furthermore, the usage of the vasoactive drug, metaraminol, in group U was lower than in group C [1 (0, 1.5) vs. 1 (0.5, 1.5)], with a mean difference of 0.283 (95% CI 0.044 to 0.522), *p* = 0.012.

**Conclusion:**

Conducting an IVC-abdominal aorta ultrasound examination before spinal anesthesia in parturients and using the IVC/Ao ratio to guide post-spinal anesthesia positioning reduced the incidence and frequency of hypotension as well as the dose of vasopressors required after surgery.

**Clinical trial registration:**

https://www.chictr.org.cn/showproj.html?proj=166587, identifier ChiCTR2200059888.

## 1 Introduction

When parturients lie in the supine position, the gravid uterus may compress the inferior vena cava (IVC). This compression can lead to a decrease in venous return to the heart. Consequently, parturients may experience hypotension, a condition known as supine hypotensive syndrome. Subarachnoid block anesthesia provides rapid and effective anesthesia for patients undergoing cesarean section (CS). However, post-spinal hypotension is exacerbated by aortocaval compression from the gravid uterus, especially when the patient is in a supine position. Spinal anesthesia induces sympathetic blockade and peripheral vasodilation, which, when combined with reduced venous return due to IVC compression, significantly increases the risk of hypotension (1–3). The prevalence of hypotension in women undergoing elective cesarean delivery ranges from 7.4 to 74.1%, depending on the definition of hypotension (4–6). However, some studies have reported that maternal hypotension following lumbar anesthesia can be as high as 35–80% (7). Kundra’s study, which used the inferior vena cava collapsibility index (IVCCI) to predict hypotension in women with a conventional left tilt of 15° following lumbar anesthesia, found an actual prevalence of post-lumbar hypotension of 76% (8).

During CS surgery, common position management strategies to mitigate the risk of hypotension in the mother following spinal anesthesia include elevating the right buttock or tilting the operating table 15° to the left (4, 9). It is believed that this strategy alleviates compression of the major abdominal vessels, specifically the IVC and abdominal aorta. This, in turn, enhances venous return, increases cardiac output and blood pressure, and reduces the incidence of hypotension after spinal anesthesia (10–12). Additionally, the 15° left lateral position is considered beneficial for improving the clinical condition of the fetus and preventing acidosis (4, 9). Guidelines for cesarean section and anesthesia-related procedures recommend that mothers maintain a 15° left lateral position before fetal delivery to reduce the incidence of hypotension after spinal anesthesia (4, 9, 13). In recent years, despite the routine use of the 15° left-tilted position for CS following spinal anesthesia, some researchers have questioned its effectiveness in reducing compression of the IVC by the gravid uterus (14–18). Currently, there is a lack of direct evidence supporting its benefits for maternal hemodynamics and uteroplacental perfusion. Furthermore, some researchers have pointed out that the left lateral position not only increases maternal discomfort but also complicates the surgical procedure (19). Therefore, controversy remains about whether the 15° left-tilting position is effective in improving maternal hemodynamic stability.

Given the ease of obtaining IVC ultrasound images with good repeatability of measurements, and the simplicity and non-invasiveness of this technique, it has become a widely utilized clinical tool for volume assessment in patients (20, 21). The collapsibility index of the IVC can, not only predict hypotension after induction of general anesthesia (22), but it can also guide volume therapy before spinal anesthesia to effectively prevent post-spinal hypotension (23). However, it should be noted that there are significant individual variations in both the measurements of the IVC diameter and collapsibility index (24). In 2008, researchers proposed using the IVC to abdominal aorta diameter (IVC:Ao diameter) as a method to assess volume status in both children and adults (25). Recent studies have revealed that preoperative evaluation of both the IVC collapsibility index and aortic index can serve as novel predictive indicators for post-spinal hypotension (26). However, the application value of the IVC:Ao diameter in obstetrics remains unclear. Therefore, in this study we hypothesized that conducting an IVC ultrasound examination before spinal anesthesia in parturients and using the IVC:Ao diameter to guide post-spinal anesthesia positioning would reduce the incidence rate of hypotension when compared to the traditional 15° left-lateral tilt.

## 2 Materials and methods

### 2.1 Trial design

This single-center, randomized controlled, non-inferiority study was conducted at Fujian Provincial Hospital. The study was approved by the Ethics Committee of Fujian Provincial Hospital (Ethical Approval No: K2021-12-063). The protocol was registered at the Chinese Clinical Trial Registry^1^ and the registration number is ChiCTR2200059888 (13/05/2022). Written informed consent was obtained from all participants. The recruitment period for observation of subjects was from May 16, 2022, to January 30, 2023. The trial was conducted according to the Declaration of Helsinki and Consolidated Standards of Reporting Trials (CONSORT) guidelines (27).

### 2.2 Patients

The inclusion criteria were: 1. Parturients with a singleton, full-term pregnancy undergoing elective CS; 2. ASA I-II; 3. Age 18–40 years; 4. Body mass index (BMI) ≤ 30 kg/m^2^. The exclusion criteria were: 1. Fetal intrauterine growth restriction, gestational age <37 weeks, height >170 cm or <150 cm, placenta previa, pre-eclampsia; 2. Pregnant women with hypertension, hyperthyroidism, kidney disease, or mental illness; 3. Women with cardiovascular disease or receiving cardiovascular medication during pregnancy; 4. Hb<90 g/L or contraindications to neuraxial anesthesia.

Exclusions of parturients while the study was in progress included: 1. Pronounced supine hypotensive syndrome during examination; 2. Refusal by the pregnant woman or her family to continue the examination; 3. Failure of neuraxial anesthesia puncture or the anesthesia plane not reaching T_4_ level, requiring alternative anesthesia; 4. Other special circumstances.

### 2.3 Randomization and blinding

Parturients were randomly assigned to two groups in a 1:1 ratio using a computer-generated number and opaque envelopes for concealing the random allocation. During spinal anesthesia, a trained anesthesiologist selected eligible parturients and opened the envelopes to determine group assignment and post-spinal anesthesia positioning. The parturients in the control group (group C) were positioned at a traditional 15° left-lateral tilt and for those in group U, the operating table was tilted to obtain the maximum IVC:Ao diameter as determined by preoperative ultrasound, which was the one with the maximum IVC:Ao diameter. The angle position of the patient must be calibrated using the protractor on a mobile telephone (Supplementary Figure 1).

A senior ultrasound physician A who was blinded to group allocation and subsequent anesthetic procedures conducted all the ultrasound examinations. Data collection and recordings were carried out by a separate ultrasound department physician B not involved in this study. The anesthesiologist A conducted both the preoperative ultrasound and post-spinal anesthesia positioning. The anesthesiologist A was not involved in anesthesia administration, data collection or recording. The group allocation was revealed only when the envelope was opened during the parturient’s lumbar anesthesia procedure, after which the patient’s position was determined according to the grouping. Another senior anesthesiologist B administered the combined spinal-epidural anesthesia and post-anesthesia medication, without knowledge of the preoperative ultrasound results and specific group assignments. Patients were kept blinded to group allocation. Data recording by nurses, blood gas analysis, Apgar scoring by physicians and statistical analysis were all carried out following a blinded approach.

### 2.4 Preoperative IVC and abdominal aorta ultrasound measurements

Upon arrival to the preoperative waiting area, parturients were placed in a separate quiet space, with the room temperature maintained at 23–25°C. After an intravenous infusion of a glucose solution and a 10-min rest in the supine position without a pillow, monitoring was conducted, including systolic blood pressure (SBP), diastolic blood pressure (DBP), pulse oximetry (SPO_2_) and heart rate (HR). A senior ultrasound physician measured the IVC and abdominal aorta in five different positions (supine, right tilt at 15° and 30°and left tilt at 15° and 30°). Ultrasound measurements were taken of the parturients’ IVC maximum diameter at the end of expiration (IVCmax), and minimum diameter at the end of inspiration (IVCmin). These and all other measurements were performed three times and averaged. The inferior vena cava collapsibility index (IVCCI) value was calculated as (IVCmax - IVCmin) / IVCmax Χ100. Parturients were instructed to relax, and the maximum diameter was measured twice during calm breathing periods to calculate the average value (IVCave). Additionally, the maximum inner diameter of the abdominal aorta (Ao) during the systolic period was measured and used to calculate the IVC:Ao diameter ratio (25, 26). Blood pressure (BP) was measured in the left upper limb when the parturients were lying in the supine position or on the left side, and in the right upper limb when they were lying on the right side. Measurements of pulse and oxygen saturation were also obtained in various positions. The individuals performing the measurements were blinded to other results as well as the parturients’ hemodynamic parameters.

Specially designed wooden positioning pads were customized to increase the accuracy of parturients’ positioning angles and mitigate potential bias induced by either the foam or other wedge-shaped pads. These wooden positioning pads were divided into 15° and 30° angles to ensure precise and replicable positioning of each parturient.

**Figure 1:**
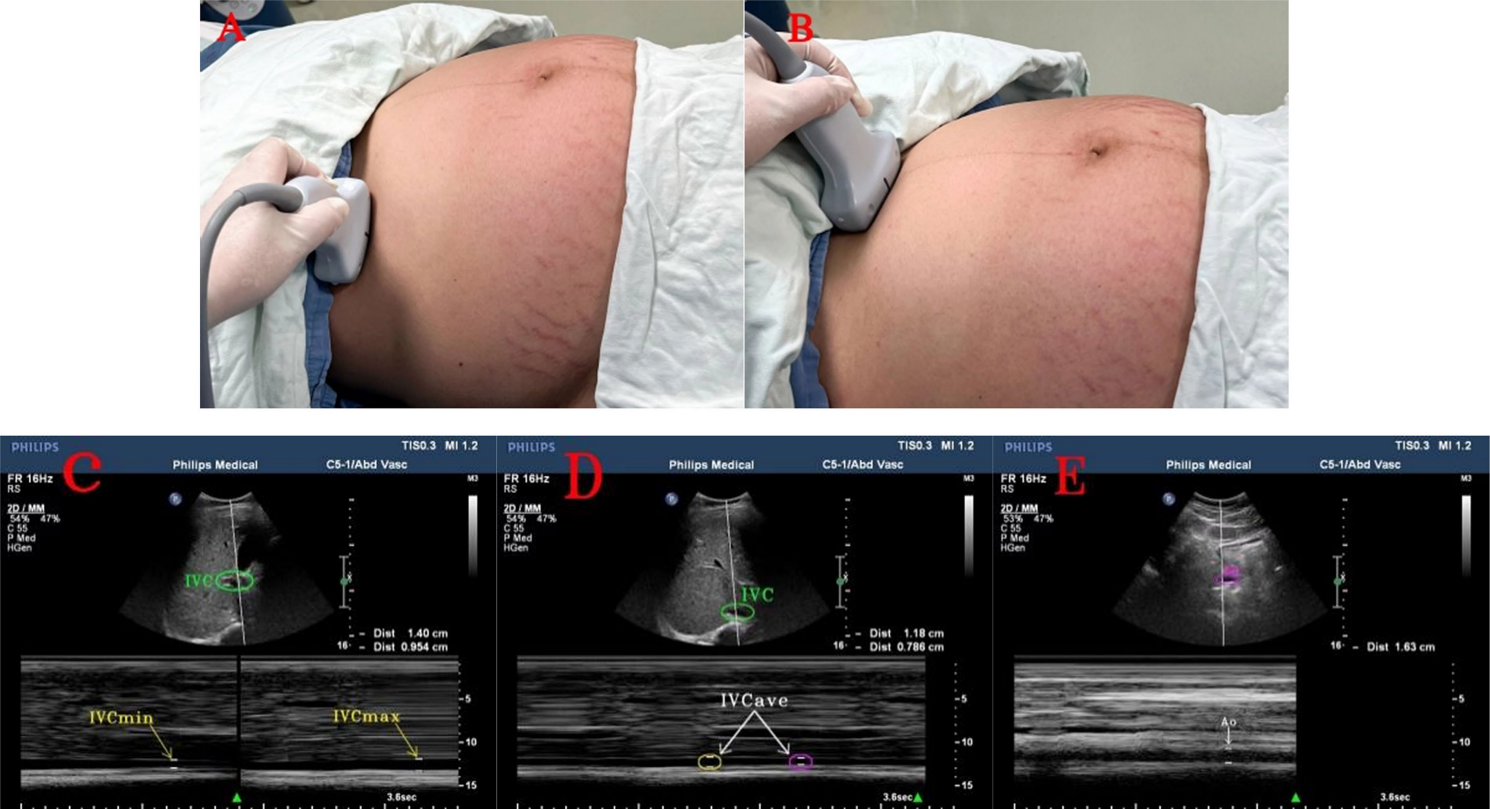
Ultrasound images of the inferior vena cava and abdominal aorta. **(A)** Ultrasound probe in the right mid-axillary line to measure the position of the inferior vena cava; **(B)** Ultrasound probe in the subxiphoid process to measure the position of the abdominal aorta; **(C)** maximum and minimum values of the inferior vena cava and inferior vena cava diameter at the end of expiration and inspiration; **(D)** two consecutive measurements of the inferior vena cava and inferior vena cava diameter during calm breathing; **(E)** abdominal aorta and abdominal aorta diameter. IVC, inferior vena cava. IVCmax, maximum value of inferior vena cava diameter at the end of expiration; IVCmin, minimum value of inferior vena cava diameter at the end of inspiration; IVCave, average value of inferior vena cava diameter on two consecutive occasions during calm breathing; Ao, abdominal aorta.

A Philips IU Elite Doppler ultrasound diagnostic device with a two-dimensional convex array abdominal probe C_5-1_, and a probe frequency of 2–5 MHz was used for all parturients. This study required ultrasonography of the inferior vena cava in five positions during late pregnancy. The final method selected was the transverse section measurement at the right mid-axillary line was based on the recommendations from previous studies on inferior vena cava ultrasonography, which was performed in multiple positions during late pregnancy (28–32). The probe was placed along the mid-axillary line, with the orientation marker pointing toward the patient’s head, and this was tilted to obtain a long axis view of the IVC passing through the liver and diaphragm. Subsequently, the probe was rotated 90° to visualize the short axis of the IVC, specifically at a point 2 cm distal to the hepatic vein’s entry into the IVC (33). This technique is applicable when the IVC remained unobservable in the subxiphoid view. The parturients were instructed to perform the Valsalva maneuver (34) at the end of expiration and inspiration, and the image from the M-mode ultrasound was to frozen used to measure the IVCmax and IVCmin. Subsequently, the parturients were instructed to relax, and the maximum inner diameter was measured twice during calm breathing periods to calculate the IVCave. The probe was placed below the central costal margin, in the subxiphoid longitudinal section, by selecting a point 5–10 mm above the abdominal aorta, and rotating the probe 90° with the orientation marker toward the patient’s right side. This measured the maximum inner diameter of the abdominal aorta during systole (25, 33) (Figure 1).

### 2.5 Anesthesia and interventions

**Figure 2:**
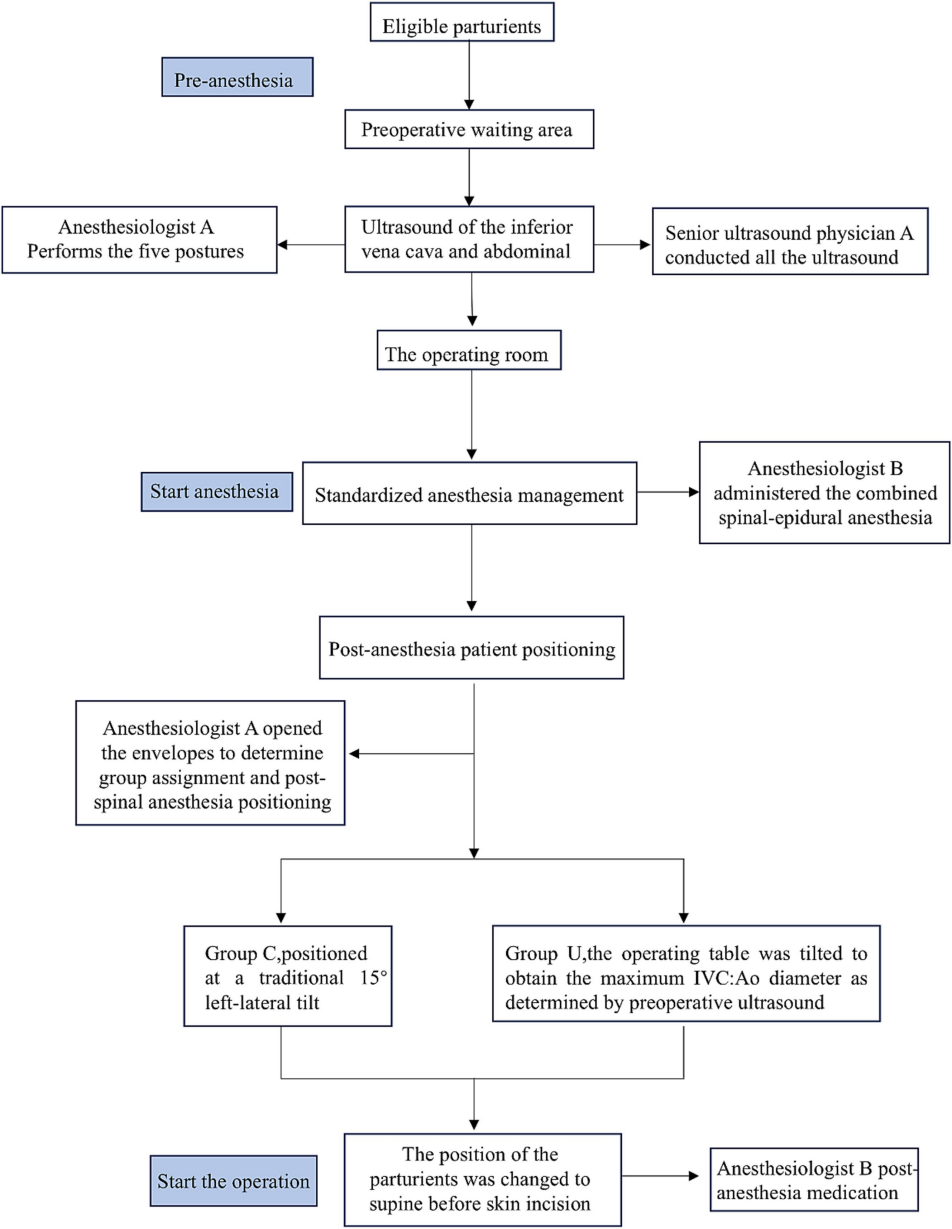
Flow chart for the protocol after the eligible parturients enter the preoperative waiting area.

All parturients fasted for more than 8 h and did not receive any medication prior to the procedure. After completing the ultrasound examination, each parturient entered the operating room and rested for 5 min before receiving 2 L/min of oxygen through a nasal cannula. A nurse, who was not involved in the study, monitored the parturients using electrocardiography (ECG), NIBP and SpO_2_. Baseline SBP, DBP and HR were measured in the supine position. The averages of three consecutive measurements of SBP, DBP, and HR were taken, with a 2-min interval between each measurement, until the difference between the three consecutive measurements and their mean values did not exceed 10%. This requirement ensured that the mother had stable BP entering the operating room. The baseline SBP was used to determine whether patients were hypotensive (SBP reduction >20% of the baseline value or SBP < 90 mm Hg) during cesarean delivery. Hypotension was defined and managed according to the guidelines outlined in the 2018 Anesthesia consensus statement on the use of vasopressors during CS under spinal anesthesia (28). Hypotension after spinal anesthesia was defined as a drop in the SBP to less than 90 mmHg, or less than 80% of the baseline value. The specific process of a parturient, from arrival at the preoperative waiting area to surgery, is illustrated in Figure 2.

All parturients received standardized anesthesia management. A senior anesthesiologist administered a combined spinal-epidural anesthesia, while the parturient was in the left lateral position. The needle-through-needle technique was used for puncturing the L_3-4_ intervertebral space. After confirming the outflow of cerebrospinal fluid (CSF), 15–17 mg of 0.7% ropivacaine diluted in CSF (adjusted according to the parturient’s height) was injected at a rate of 10 s per mL. Following the spinal needle withdrawal, an epidural catheter was inserted after ensuring no blood or CSF was withdrawn, and then it was flushed with 3 mL of saline without giving a test dose of epidural medication. The end of the spinal injection was marked as time A.

During placement of the neuraxial blockade, a researcher opened the envelope to determine the group that was assigned to the patients and then performed the post-spinal anesthesia positioning.

The sensory block level was checked by using a pinprick test 2 min after the spinal injection and again at 4, 6 and 8 min. If the sensory block level did not reach the T_4_ dermatome before the skin incision, the patient was excluded from the study.

If the patient experienced pain before delivery, 3 mL of 2% lidocaine was administered via the epidural catheter, which was removed after the surgery was completed. In order to not affect the surgical procedure, as in routine operations, the position of the patient was changed to supine before skin incision. We recorded the time they were placed in the prone position as time B.

After spinal anesthesia, BP was measured every 2 min instead of every 3 min until the baby was born. In cases of hypotension after spinal anesthesia, manifested as dizziness, difficulty breathing, nausea or vomiting, 0.5 mg of metaraminol or 6 mg of ephedrine was administered based on the patients’ HR. Ephedrine was given if the HR was below 50 bpm and if it was above 50 bpm metaraminol was administered. If the HR dropped below 50 bpm without hypotension, 0.5 mg of atropine was used.

### 2.6 Outcome measures

The primary endpoint was the incidence of hypotension in parturients, defined as the period from the completion of intrathecal drug injection (time A) to when they were placed in the supine position (time B). The secondary endpoints included the total use of vasopressors, umbilical cord blood parameters such as the pH and base excess (BE) values of the umbilical artery (UA) immediately after the birth, Apgar scores at 1 and 5 min post-birth, obstetric surgeon satisfaction and the number of hypotension incidents in parturients (from the end of spinal anesthesia until supine position). The surgeon satisfaction was based on a scale of 1–4 which was defined as 1: highly satisfied, 2: satisfied, 3: neutral and 4: dissatisfied.

### 2.7 Sample size

The primary outcome of our study was to reduce the incidence of hypotension after spinal anesthesia. Our hypothesis was that the ultrasound-guided group would demonstrate non-inferiority in preventing post-spinal hypotension when compared to the traditional 15° left tilt. Our preliminary experiments found that the incidence of post-spinal hypotension in parturients with the traditional 15° left tilt was about 66%, while it was about 58% in the ultrasound-guided group. Therefore, we assumed a one-sided alpha of 0.05, a power of 1-*β* and 0.9, and a pre-specified non-inferiority delta of 15% to assess the non-inferiority of the ultrasound-guided group. Using PASS software, version 11.0 (NCSS) for sample size calculation, we determined that each group needed 77 participants. Taking into account a dropout rate of 20% attributed to reasons such as withdrawal, we finally determined a sample size of 93 participants per group. Consequently, a total of 200 parturients were recruited for the study.

### 2.8 Statistical analysis

Statistical analyses for primary and secondary outcomes were performed using the SPSS 25.0 software package (IBM Corp, Chicago, IL, USA) and R, version 4.0.3. Missing data, which accounted for less than 5% of the total, were handled using multiple imputation (MI). Quantitative data were presented as means ± standard deviations or medians (interquartile ranges). Categorical data were expressed as [number (%)]. *p* < 0.05 was considered statistically significant. All data were tested for normality by using the Shapiro–Wilk test. Normally distributed variables for comparison between groups U and C at baseline were analyzed using the *t*-test and non-normally distributed ones were analyzed using the Mann–Whitney U test. Categorical variables were analyzed using the chi-square test. A generalized estimating equation was used to detect significant differences in repeated hemodynamic parameters and measure trends over time for each measured variable. IVC values were expressed as means ± standard deviations and compared using the independent samples *t*-test for differences in various tilt positions, with uncorrected and corrected data adjusted using the Bonferroni method. To assess non-inferiority in the primary outcome of hypotension incidence, we calculated the risk difference (RD) between the ultrasound-guided group (Group U) and the control group (Group C), along with its 95% confidence interval (CI). Non-inferiority was considered established if the lower bound of the CI was greater than the pre-defined non-inferiority margin of −15%. Additionally, a two-sample *Z*-test for proportions was performed to evaluate the statistical significance of between-group differences. For secondary outcomes, normally distributed variables were analyzed using the *t*-test, while non-normally distributed ones were analyzed using the Mann–Whitney U test.

## 3 Results

### 3.1 Participant flow and baseline data

**Figure 3:**
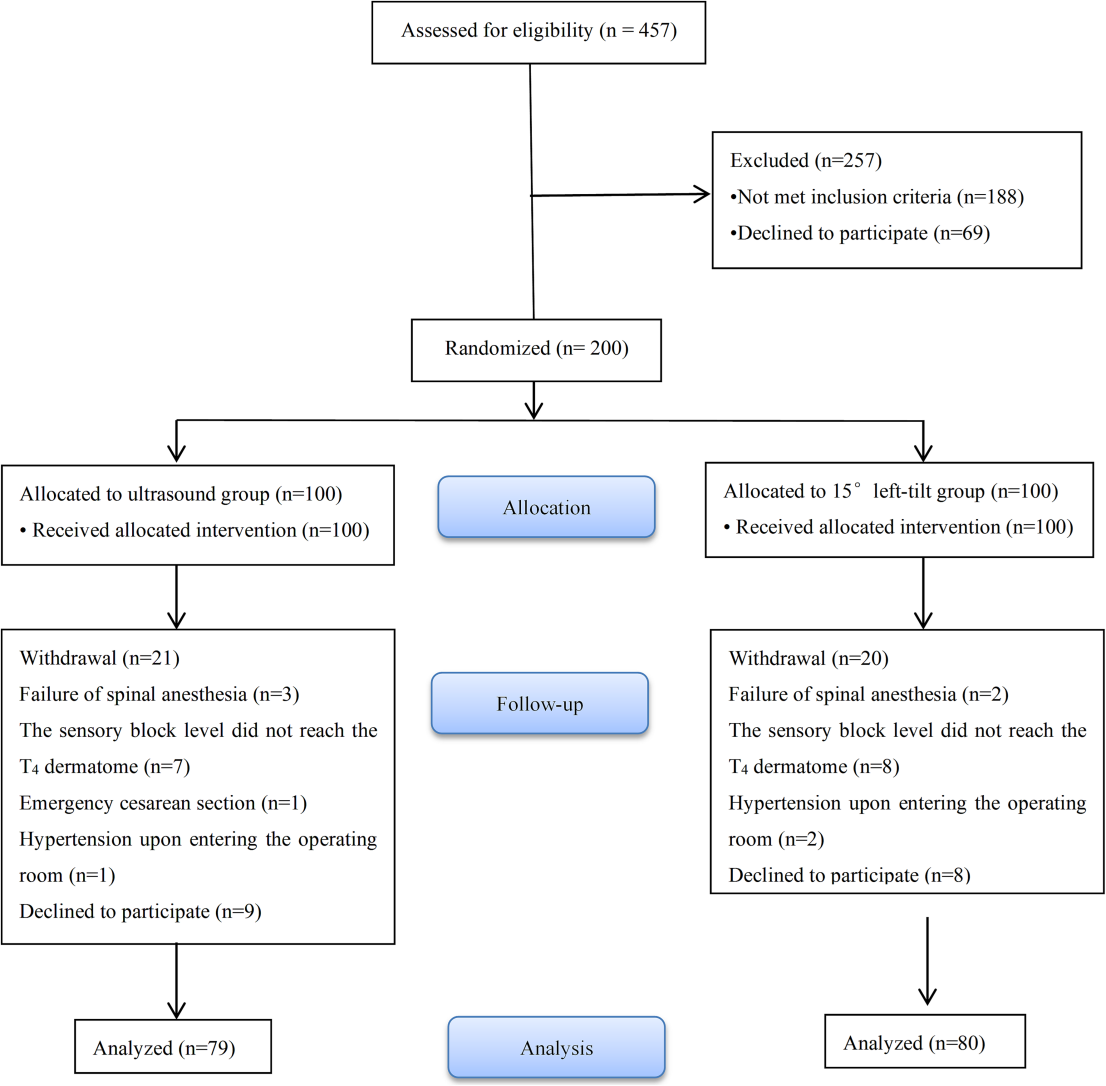
Flow diagram of the study participants.

457 parturients were recruited from May 16, 2022 to January 30, 2023. Among these, 49 parturients refused to participate in the study and 188 were excluded for not meeting the inclusion criteria. 200 parturients were randomly assigned to groups U (*n* = 100) and C (*n* = 100). In groups U and C, 21 and 20 parturients were excluded from the study and this resulted in 79 and 80 parturients, respectively, being included in the analysis. The detailed information of the participants is provided in Figure 3.

There were no significant differences in preoperative baseline data (Table 1) and ultrasound measurements between the two groups (Supplementary Table 1).

**Table 1:** Baseline characteristics of parturients in Group U and Group C.

	Group U (*n* = 79)	Group C (*n* = 80)	*p*-value
Age (years)	30.9 ± 4.0	31.9 ± 4.0	0.118
Weight (kg)	67.9 ± 7.8	68.0 ± 7.1	0.891
Primiparous or not			
Yes	28 (35.4)	26 (32.5)	0.695
No	51 (64.6)	54 (67.5)	
Pre-pregnancy (kg)	54.0 ± 6.5	56.2 ± 8.7	0.073
Height (cm)	160.2 ± 3.9	160.9 ± 4.0	0.277
BMI (kg/m^2^)	26.4 ± 2.6	26.3 ± 2.5	0.754
Gestation (weeks)	38.7 ± 0.8	38.7 ± 1.4	0.922
Abdominal Circumference (cm)	99.2 ± 13.9	99.8 ± 11.8	0.763
Fundal height (cm)	37.5 ± 12.7	36.6 ± 10.4	0.622
HGB (g/L)	124.7 ± 9.8	127.2 ± 10.5	0.117

Data are reported as mean±SD or number (%). Continuous data comparisons between Groups U and C were analyzed using the *t*-test, and categorical data were analyzed using chi-square test. BMI, body mass index; HGB, hemoglobin.

### 3.2 Primary outcome

The primary outcome measure is shown in Table 2. The incidence of hypotension from the end of spinal anesthesia until lying in the supine position was lower in the U group [48 (60.8%)] compared to the C group [64 (80%)], with a difference in incidence rate Risk Difference (RD) = −0.192, 95% CI (−0.325 to −0.050), *p* = 0.010. With respect to positional analysis, in group U there were 7 (8.86%) parturients in the supine position, 17 (21.52%) in the left 15° position, 19 (24.05%) in the left 30° position, 9 (11.39%) in the right 15° position and 27 (34.18%) in the right 30° position.

**Table 2:** Incidence of hypotension before supine positioning and associated risk difference.

	Group U (*n* = 79)	Group C (*n* = 80)	RD Difference in means (95% CI)	*p*-value
Hypotension occurring before supine positioning	48 (60.8)	64 (80.0)	−0.192 (−0.325,-0.050)	**0.010**

Data are reported as number (%). The primary endpoint parameters were assessed using risk difference and *Z*-test. RD, risk difference. Bold was used to indicate *p*-values which were significant (<0.05).

### 3.3 Secondary outcomes and other outcome

The use of the vasopressor, metaraminol, was lower in group U compared to the group C (1 (0, 1.5) vs. 1 (0.5, 1.5), MD 0.283 (95% CI 0.044 to 0.522); *p* = 0.012). Ephedrine use was observed only in group C, with two parturients using 6 and 12 mg, respectively, and none in group U (0 (0%) vs. 2 (2.5%); *p* = 0.245). No parturients in either group used atropine (0 (0%) vs. 0 (0%); *p* = 1) (Table 3).

**Table 3:** Secondary outcome measures, drug use, and neonatal parameters in two groups.

	Group U (*n* = 79)	Group C (*n* = 80)	Mean difference (95% CI)	*p*-value
Umbilical artery pH	7.32 ± 0.04	7.32 ± 0.05	0.01 (−0.01, 0.02)	0.397
Obstetric satisfaction value				
1	79 (100)	80 (100)	/	1.000
2	0 (0)	0 (0)		
3	0 (0)	0 (0)		
4	0 (0)	0 (0)		
BE value (mmol/L)	−0.29 ± 1.46	−0.42 ± 1.75	0.13 (−0.63, 0.37)	0.605
1-Minute Apgar Score	9.9 ± 0.3	9.9 ± 0.3	0.0 (−0.1, 0.1)	0.778
5-Minute Apgar Score	10.0 ± 0.2	9.9 ± 0.2	0.0 (−0.1, 0.0)	0.255
Metaraminol (mg)	1 (0, 1.5)	1 (0.5, 1.5)	0.3 (0.0, 0.5)	**0.012**
Ephedrine use, *n* (%)	0 (0%)	2 (2.5%)	/	0.245
Atropine use, *n* (%)	0 (0%)	0 (0%)	/	1.000
Anesthesia duration (min)	5.7 ± 2.8	5.6 ± 2.5	0.1 (−1.0, 0.8)	0.882
Time from end of anesthesia to skin incision (min)	8.7 ± 2.5	8.6 ± 2.4	0.0 (−0.8, 0.8)	0.329
Surgical duration (min)	76.5 ± 12.1	78.1 ± 10.2	−3.8 (−7.4, 0.1)	0.359
Time from end of anesthesia to delivery (min)	19.6 ± 4.1	20.1 ± 8.0	0.3 (−0.9, 1.5)	0.661
Pre-anesthesia fluid administration (mL)	499 ± 43	499 ± 35	0 (−12, 12)	0.999
Fluid administration at end of anesthesia (mL)	603 ± 51	603 ± 38	0 (−14, 14)	0.996
Fluid administration at time of fetal extraction (mL)	885 ± 80	899 ± 118	−15 (−46, 17)	0.364
Blood loss (mL)	380 ± 131	356 ± 67	24 (−8, 57)	0.146
Total Output(ml)	887 ± 144	860 ± 86	27 (−10, 64)	0.157
Fetal weight (g)	3,443 ± 430	3,351 ± 4,223	93 (−41, 226)	0.173

Data are reported as mean  ±  SD, number (%) or median (interquartile range), as appropriate. Continuous data comparisons between Group U and Group C were analyzed using the *t*-test or Mann–Whitney U test depending on data distribution. Categorical variables were analyzed using the chi-square test or Fisher’s exact test, as appropriate. Bold was used to indicate *p*-values which were significant (<0.05). BE Value, base excess value at birth.

There were no significant differences between the two groups in the immediate umbilical artery pH values after birth (7.32 ± 0.04 vs. 7.32 ± 0.05, MD 0.01 [95% CI −0.01 to 0.02]; *p* = 0.397), BE values (−0.29 ± 1.46 vs. −0.42 ± 1.75, MD 0.13 [95% CI −0.63 to 0.37]; *p* = 0.605), as well as in the Apgar scores at 1 and 5 min after birth. The satisfaction assessment for obstetric surgeons in both groups was 100% (79 (100%) vs. 80 (100%); *p* = 1) (Table 3).

No significant differences were observed between the two groups in pre-anesthesia fluid administration (499 ± 43 vs. 499 ± 35, MD 0 [95% CI −12 to 12]; *p* = 0.999), fluid administration at the end of anesthesia (603 ± 51 vs. 603 ± 38, MD 0 [95% CI −14 to 14]; *p* = 0.996) and fluid administration at the time of fetal extraction (855 ± 80 vs. 899 ± 118, MD −15 [95% CI −46 to 17]; *p* = 0.364; Table 3).

**Figure 4:**
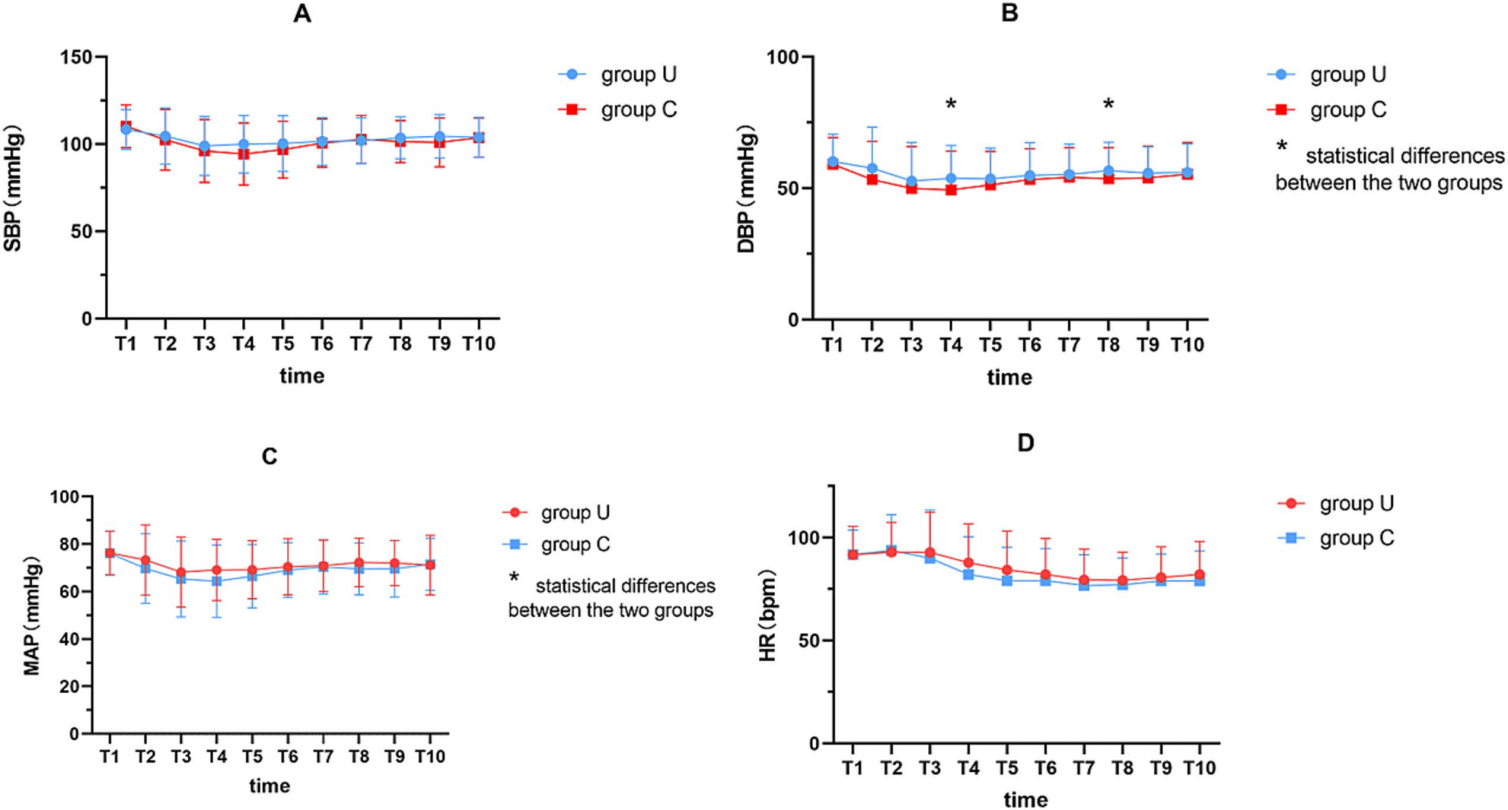
Perioperative hemodynamic variables indicators. **(A)** SBP; **(B)** DBP; **(C)** MAP; **(D)** HR. A generalized estimation equation to analyze the hemodynamics at 18 min post-anesthesia. T1, immediately after spinal anesthesia; T2, T3, T4, T5, T6, T7, T8, T9 and T10 at 2, 4, 6, 8, 10, 12, 14, 16 and 18 min after spinal anesthesia, respectively. * represents *p* < 0.05. SBP, systolic blood pressure; DBP, diastolic blood pressure; MAP, mean arterial pressure; HR, heart rate.

A generalized estimating equation for analyzing the hemodynamics at 18 min post-anesthesia (Figure 4; Supplementary Table 2) found no significant interaction in SBP between the groups (*F* = 1.96, *p* = 0.163), although there were significant changes over time (*F* = 13.19, *p* < 0.001). Additionally, there was a significant interaction between the DBP (*F* = 4.13, *p* = 0.044) and changes over time (*F* = 8.08, *p* < 0.001). Six minutes after spinal injection, the DBP in group U was significantly higher than in group C (54 ± 13, 49 ± 15; *p* = 0.019). Furthermore, no significant interaction was found in MAP (*F* = 3.38, *p* = 0.068), but there were significant changes over time (*F* = 10.70, *p* < 0.001). In addition, there was no significant interaction in pulse rate between the groups (*F* = 2.08, *p* = 0.151), but significant changes were observed over time (*F* = 36.28, *p* < 0.001).

## 4 Discussion

This trial is the first to explore the difference of using of pre-spinal anesthesia IVC ultrasound examination and the IVC:Ao diameter to guide post-spinal anesthesia positioning in parturients, when compared with the traditional 15° left-lateral tilt in a non-inferiority, randomized controlled trial. We concluded that using the IVC:Ao diameter to guide post-spinal anesthesia positioning significantly reduced the incidence and frequency of hypotension as well as the required dosage of vasopressors when compared to the traditional method during surgical preparation.

A Cochrane analysis of 11 studies involving 857 parturients undergoing CS found no effect of using the left-lateral tilt when compared to the horizontal position on either the SBP or the incidence of hypotensive episodes. Furthermore, no significant differences in maternal HR, 5-min Apgar scores and umbilical cord pH values (17). Moreover, our study revealed no significant differences in 1- and 5-min Apgar scores, umbilical cord pH values and the obstetricians’ satisfaction between using the IVC:Ao diameter for post-spinal anesthesia positioning and the 15° left tilt. Parturients experience a rapid decrease in systemic vascular resistance (SVR) and BP between 1 and 6 min after spinal medication (35), as was confirmed by our study. Six minutes after spinal injection, we observed significantly improved hemodynamics in parturients from group U, as compared to those in group C. Ultrasound measurements of the IVC:Ao diameter have been introduced into clinical practice to assess intravascular volume, and it is has been reported to be a reliable, non-invasive and simple technique for assessing volume status. The IVC:Ao diameter has also been shown to assess volume status in children and adults (25), and it is considered to act as a novel predictor of post-spinal hypotension with higher sensitivity, specificity and accuracy over than the IVCCI (26). Ultrasonographic evaluation of the IVC and the internal jugular vein (IJV), and measurement of the dynamic variation in diameter facilitates calculation of a collapsibility index (CI) to predict the hypotensive response to spinal anesthesia. Both IVC-CI and IJV-CI are reliable markers with a sensitivity and specificity greater than 80% for predicting post spinal anesthesia hypotension (36). Our study indicated that preoperative ultrasound examination of IVC:Ao diameter to guide post-spinal anesthesia positioning can better reduce the incidence of hypotension compared to group C during surgical preparation, which is consistent with previous findings that a 30° left tilt significantly reduces the incidence of hypotension (16).

Which is the best position to reduce post-spinal hypotension in parturients? Currently, it has been shown that the IVC is completely compressed in full-term pregnant women in the supine position, and a 30° left tilt, partially relieves this compression (13, 37). In an MRI study of pregnant women in their third trimester, the supine position resulted in a 16.4% decrease in cardiac output compared with the left lateral position and a 32% reduction of blood flow through the abdominal aorta at the level of its bifurcation (38). Another study observed that approximately 25% of parturients exhibited a larger maximum diameter of the IVC when they were subjected to a right tilt position, when compared to the supine and left tilt positions (29). A study utilizing magnetic resonance imaging technology revealed that a 30° left tilt position effectively relieved compression of the IVC caused by the gravid uterus in comparison to the supine position. However, for some parturients, a 30° right tilt position could optimize the vena cava volume (39). The reasons for changes in the vena cava volume with different positions are unclear, and the degree of compression by the gravid uterus can be influenced by multiple factors (40). Recently, Chiara Sonnino and colleagues observed that cardiac output did not decrease significantly after a 15° left uterine displacement removal in patients under spinal anesthesia for CS during continuous hemodynamic monitoring (41). In another study, a noninvasive cardiac output monitoring system was used to compare the left-tilt and supine positions in full-term parturients under spinal anesthesia with respect to haemodynamics. They observed that the haemodynamic parameters were not statistically different between the two groups. The incidence of nausea and vomiting was also not significantly different between the two groups (42). In our study, only 17 parturients (21.52%) in group U were in a 15° left tilt Therefore, our study suggested that using a 15° left tilt post-spinal anesthesia may not provide hemodynamic benefits for all parturients, as some may find other positions more suitable. Our findings may partially explain the contradictory results regarding the beneficial effects of the right tilt on maternal hemodynamics and support case reports showing where only a right tilt was effective for supine hypotensive syndrome in women undergoing CS (20, 21).

In our study, the incidence of hypotension before skin incision in group C was 77.5% without the simultaneous use of vasopressors, which was similar to previous studies showing 72% (16). Obstetricians consider a 15° left tilt to be a significant obstacle to surgical conditions, commonly requesting its reduction to less than 10 degrees before the start of surgery (43). Therefore, a strict 15° left tilt is rarely maintained until fetal delivery during routine clinical practice. Consequently, our study did not adhere to maintaining the same position until the fetus was delivered, intervening only during surgical preparation, thereby enhancing the generalizability of our findings in clinical practice (43).

This study had some limitations. Firstly, although all ultrasound measurements in this study were performed by a dedicated ultrasound physician, and great care was taken when positioning parturients, the accuracy of every angle used could not be guaranteed. Secondly, Secondly, due to the significant hypotension that can occur shortly after spinal anesthesia in pregnant women, we are currently unable to continue using ultrasound to measure the compression of the IVC and abdominal aorta in the five different positions without intervention. We also cannot guarantee IVC:Ao ratio measurements before hypotension occurs. This is a study limitation. After spinal anesthesia in pregnant women, it is important that the procedure does not affect the surgeon’s ability to perform the cesarean section, while also allowing continuous monitoring of the inferior vena cava and abdominal aorta in different maternal positions. Perhaps portable, real-time ultrasound monitoring could complete these observational studies (44). Thirdly, our study subjects had a BMI ≤ 30 kg/m^2^, and others have shown that BMI can affect the effectiveness of left tilt (34). Finally, this study was conducted as a single-center and non-inferiority design. Future studies should include multicenter randomized controlled trial. Therefore, the clinical significance of our results needs to be verified in future studies from multiple perspectives.

In conclusion, we found that using pre-spinal anesthesia IVC ultrasound examination and the IVC:Ao diameter to guide post-spinal anesthesia positioning in parturients significantly reduced the incidence and frequency of hypotension when compared to the traditional 15° left tilt. In addition, the total dosage of vasopressors required was also reduced.

## Data Availability

The datasets presented in this study can be found in online repositories. The names of the repository/repositories and accession number(s) can be found in the article/Supplementary material.

## Footnotes

^1^ https://www.chictr.org.cn

## Supplementary material

SUPPLEMENTARY FIGURE 1 Posture placement after spinal anesthesia: the posture angle must be calibrated using the protractor on a mobile telephone.
