# Supplementary figures and images for: Preoperative inferior vena cava-abdominal aorta ultrasound examination to guide the positioning of spinal anesthesia to reduce post-spinal hypotension: a prospective, randomized trial
Source: Front Med (Lausanne). 2025 Oct 9;12:1641899. doi: 10.3389/fmed.2025.1641899 (PMC12548758; doi:10.3389/fmed.2025.1641899)

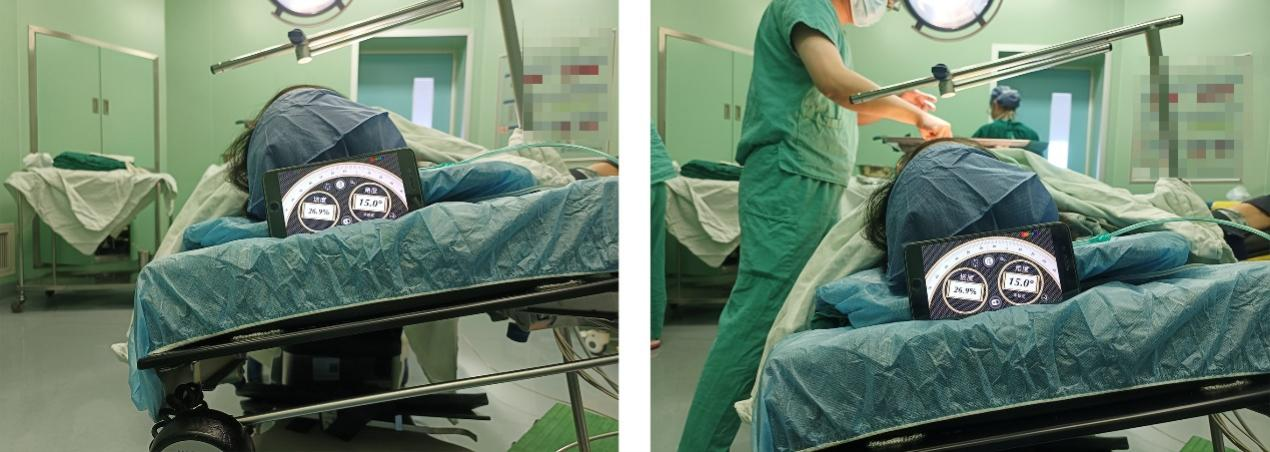

Supplement: SUPPLEMENTARY FIGURE 1 — Posture placement after spinal anesthesia: the posture angle must be calibrated using the protractor on a mobile telephone. [file Image_1.TIF]
