# Supplementary material for: Preoperative inferior vena cava-abdominal aorta ultrasound examination to guide the positioning of spinal anesthesia to reduce post-spinal hypotension: a prospective, randomized trial
Source: Front Med (Lausanne). 2025 Oct 9;12:1641899. doi: 10.3389/fmed.2025.1641899 (PMC12548758; doi:10.3389/fmed.2025.1641899)
Supplement: Supplementary file 2 [file Table_1.doc]

**Supplementary Table 1：** Ultrasound data of parturients

|  | Group U (n=79) | Group C (n=80) | *P*-value |
| --- | --- | --- | --- |
| Baseline |  |  |  |
| SBP | 114±10 | 116±11 | 0.225 |
| DBP | 70±8 | 71±9 | 0.496 |
| MAP | 85±8 | 86±9 | 0.331 |
| HR | 87±11 | 89±13 | 0.310 |
| SpO_2_ | 98±1 | 97±4 | 0.336 |
| Supine |  |  |  |
| SBP | 112±11 | 112±11 | 0.614 |
| DBP | 69±8 | 70±9 | 0.556 |
| MAP | 83±8 | 84±9 | 0.546 |
| HR | 86±12 | 88±14 | 0.221 |
| SpO_2_ | 98±1 | 98±1 | 0.962 |
| IVCmin | 11.2±3.1 | 10.6±2.8 | 0.264 |
| IVCmax | 14.8±3.2 | 14.2±2.9 | 0.198 |
| IVCCI | 25.3±10.9 | 25.2±11.9 | 0.969 |
| IVCave | 11.6±2.9 | 11.6±2.8 | 0.845 |
| Ao | 14.8±1.8 | 14.7±2.2 | 0.654 |
| IVC:Ao | 0.8±0.2 | 0.8±0.5 | 0.433 |
| Left 15°tilt |  |  |  |
| SBP | 114±10 | 116±10 | 0.270 |
| DBP | 68±8 | 71±9 | 0.096 |
| MAP | 83±8 | 86±9 | 0.107 |
| HR | 86±10 | 87±11 | 0.492 |
| SpO_2_ | 98±1 | 98±2 | 0.743 |
| IVCmin | 11.4±2.7 | 10.9±2.9 | 0.364 |
| IVCmax | 15.2±3.0 | 14.7±2.8 | 0.271 |
| IVCCI | 25.2±10.5 | 25.8±10.9 | 0.716 |
| IVCave | 12.3±2.5 | 12.1±2.8 | 0.763 |
| Ao | 14.9±1.7 | 15.0±1.5 | 0.646 |
| IVC:Ao | 0.8±0.2 | 0.8±0.2 | 0.522 |
| Left 30°tilt |  |  |  |
| SBP | 113±10 | 114±10 | 0.505 |
| DBP | 68±8 | 69±8 | 0.268 |
| MAP | 83±7 | 84±8 | 0.278 |
| HR | 86±9 | 88±12 | 0.181 |
| SpO_2_ | 97±3 | 97±2 | 0.944 |
| IVCmin | 11.5±2.7 | 11.0±3.4 | 0.329 |
| IVCmax | 15.1±2.5 | 14.7±3.2 | 0.364 |
| IVCCI | 24.3±9.8 | 25.8±11.9 | 0.407 |
| IVCave | 12.6±2.5 | 12.3±2.9 | 0.475 |
| Ao | 15.2±1.6 | 15.2±1.5 | 0.973 |
| IVC:Ao | 0.8±0.2 | 0.8±0.2 | 0.440 |
| Right 15°tilt |  |  |  |
| SBP | 113±9 | 115±11 | 0.160 |
| DBP | 69±7 | 71±9 | 0.075 |
| MAP | 84±7 | 86±9 | 0.072 |
| HR | 88±11 | 89±13 | 0.596 |
| SpO_2_ | 98±1 | 98±1 | 0.988 |
| IVCmin | 11.3±2.9 | 11.6±2.7 | 0.504 |
| IVCmax | 15.0±2.9 | 15.4±3.0 | 0.315 |
| IVCCI | 25.1±10.2 | 25.0±10.0 | 0.935 |
| IVCave | 12.3±2.7 | 12.6±2.8 | 0.480 |
| Ao | 14.9±2.2 | 15.3±1.7 | 0.138 |
| IVC:Ao | 0.9±0.9 | 0.8±0.2 | 0.359 |
| Right 30°tilt |  |  |  |
| SBP | 114±9 | 115±11 | 0.352 |
| DBP | 67±8 | 69±9 | 0.085 |
| MAP | 83±7 | 85±9 | 0.119 |
| HR | 86±11 | 89±12 | 0.222 |
| SpO_2_ | 98±1 | 98±1 | 0.615 |
| IVCmin | 11.8±2.9 | 11.7±3.1 | 0.835 |
| IVCmax | 15.6±3.0 | 15.6±3.0 | 0.980 |
| IVCCI | 24.2±10.2 | 25.1±11.0 | 0.593 |
| IVCave | 12.8±2.9 | 13.0±3.1 | 0.642 |
| Ao | 15.2±2.0 | 15.3±1.6 | 0.950 |
| IVC:Ao | 0.9±0.2 | 0.9±0.2 | 0.877 |

Data are reported as mean ± SD. Continuous data comparisons between Groups U and C were analyzed using the t-test. SBP, systolic blood pressure; DBP, diastolic blood pressure; MAP, mean arterial pressure; HR, heart rate; SpO_2_, pulse oxygen saturation; IVCmin, minimum value of inferior vena cava diameter at the end of inspiration; IVCmax, maximum value of inferior vena cava diameter at the end of expiration; IVCCI,(IVCmax- IVCmin)/IVCmax* 100; IVCave, average value of inferior vena cava diameter on two consecutive occasions during calm breathing; Ao, abdominal aorta.
