# Supplementary material for: Preoperative inferior vena cava-abdominal aorta ultrasound examination to guide the positioning of spinal anesthesia to reduce post-spinal hypotension: a prospective, randomized trial
Source: Front Med (Lausanne). 2025 Oct 9;12:1641899. doi: 10.3389/fmed.2025.1641899 (PMC12548758; doi:10.3389/fmed.2025.1641899)
Supplement: Supplementary file 3 [file Table_2.doc]

**Supplementary Table 2: Perioperative hemodynamic variables**

|  | Group U (n=79) | Group C (n=80) | *P*-value |
| --- | --- | --- | --- |
| SBP |  |  |  |
| T1 | 108 ± 12 | 110 ± 12 | 0.334 |
| T2 | 105±16 | 102±17 | 0.297 |
| T3 | 99±17 | 96±18 | 0.292 |
| T4 | 100 ± 17 | 94 ± 18 | 0.041 |
| T5 | 100±16 | 97±16 | 0.095 |
| T6 | 102±14 | 101±14 | 0.75 |
| T7 | 102 ± 13 | 103± 14 | 0.753 |
| T8 | 104 ± 12 | 102 ± 12 | 0.288 |
| T9 | 104±12 | 101±14 | 0.067 |
| T10 | 104 ± 11 | 104 ± 11 | 0.924 |
|  |  | F | *P* |
|  | time | 13.19 | <0.001 |
|  | group | 1.96 | 0.163 |
|  | time*group | 1.20 | 0.288 |
| DBP |  |  |  |
| T1 | 60±10 | 59±10 | 0.533 |
| T2 | 58±16 | 53±15 | 0.072 |
| T3 | 53±15 | 50±16 | 0.203 |
| T4 | 54±13 | 49± 15 | **0.019** |
| T5 | 54 ± 12 | 51 ± 13 | 0.23 |
| T6 | 55±13 | 53±12 | 0.535 |
| T7 | 55 ± 11 | 54 ± 11 | 0.536 |
| T8 | 57±11 | 54±12 | **0.035** |
| T9 | 56±10 | 54±12 | 0.12 |
| T10 | 56 ± 11 | 55 ± 12 | 0.716 |
|  |  | F | *p* |
|  | time | 8.08 | <0.001 |
|  | group | 4.13 | 0.044 |
|  | time*group | 0.61 | 0.792 |
| MAP |  |  |  |
| T1 | 76±9 | 76±9 | 0.927 |
| T2 | 73±15 | 70±15 | 0.103 |
| T3 | 68±15 | 65±16 | 0.238 |
| T4 | 69±13 | 64±15 | 0.01 |
| T5 | 69±12 | 66±13 | 0.077 |
| T6 | 70 ± 12 | 69±12 | 0.454 |
| T7 | 71 ± 11 | 70±11 | 0.77 |
| T8 | 72 ± 10 | 70±11 | 0.108 |
| T9 | 72±10 | 70±12 | 0.042 |
| T10 | 71±13 | 71±11 | 0.808 |
|  |  | F | *p* |
|  | time | 10.70 | <0.001 |
|  | group | 3.38 | 0.068 |
|  | time*group | 0.93 | 0.495 |
| HR |  |  |  |
| T1 | 92 ± 14 | 92 ± 12 | 0.979 |
| T2 | 93 ± 15 | 94 ± 17 | 0.741 |
| T3 | 93 ± 20 | 90 ± 23 | 0.42 |
| T4 | 88±19 | 82±18 | 0.075 |
| T5 | 84±19 | 79±16 | 0.074 |
| T6 | 82±17 | 79±16 | 0.237 |
| T7 | 79±15 | 77±15 | 0.134 |
| T8 | 79±14 | 77±13 | 0.212 |
| T9 | 81±15 | 79±13 | 0.621 |
| T10 | 82±16 | 79±15 | 0.218 |
|  |  | F | *p* |
|  | time | 36.28 | <0.001 |
|  | group | 2.08 | 0.151 |
|  | time*group | 1.16 | 0.318 |

Data are reported as mean±SD. A generalized estimating equation analyzing the hemodynamics at 18 minutes post-anesthesia. T1, immediately after spinal anesthesia; T2, T3, T4, T5, T6, T7, T8, T9 and T10 at 2, 4, 6, 8, 10, 12, 14, 16 and 18 minutes after spinal anesthesia, respectively. Bold was used to indicate p-values which were significant (<0.05). SBP, systolic blood pressure; DBP, diastolic blood pressure; MAP, mean arterial pressure; HR, heart rate.
